# Green Tea Polyphenol Epigallocatechin-3-Gallate Promotes Reendothelialization in Carotid Artery of Diabetic Rabbits by Reactivating Akt/eNOS Pathway

**DOI:** 10.3389/fphar.2018.01305

**Published:** 2018-11-14

**Authors:** He Huang, Chong-ying Jin, Xu-kun Bi, Yan-bo Zhao, Sheng-jie Xu, Mei-hui Wang, Lu Yu, Ya-xun Sun, Dan Hu

**Affiliations:** ^1^Department of Cardiology, Sir Run Run Shaw Hospital, College of Medicine, Zhejiang University, Hangzhou, China; ^2^Department of Cardiology and Cardiovascular Research Institute, Renmin Hospital of Wuhan University, Wuhan, China; ^3^Hubei Key Laboratory of Cardiology, Wuhan, China

**Keywords:** EGCG, L-EPC, diabetes, reendothelialization, proliferation, migration, Akt, eNOS

## Abstract

**Background:** Epigallocatechin gallate (EGCG) is the most abundant catechin in green tea and has proven benefits on endothelial cells in diabetes. However, it remains unclear whether EGCG could improve function of late endothelial progenitor cells (L-EPCs) in diabetes.

**Methods:** Thirty-six rabbits were randomized into six groups. Thirty diabetic rabbits were induced by a single dose of alloxan (100 mg/kg injection intraperitoneally). All of them were given intragastrically EGCG (50 mg/kg/day) or saline for 7 days after carotid injury. In autotransfusion experiment, L-EPCs were cultured with pre-treated EGCG (40 μM for 72 h) and then were injected into the site of injured vascular. Proliferation and migration of EGCG pre-treated L-EPCs in high glucose condition were assessed by EDU incorporation assay and modified Boyden chamber assay, respectively. The mRNA and protein expression of Akt-eNOS pathway were detected by real-time PCR and western blot.

**Results:** Reendothelialization rate in injured carotid artery of diabetic rabbits was augmented in the EGCG group (50 mg/kg/d for 7 days) compared with the non-EGCG group (74.2 ± 4.6% vs. 25.6 ± 5.9%, *P* < 0.001). EGCG pre-treated L-EPCs autologous transfusion also accelerated the diabetic rabbits’ carotid reendothelialization compared with the diabetic sham-operated group (65.6 ± 8.5% vs. 32.9 ± 5.0%, *P* = 0.011). *In vitro* studies showed, 40 μM EGCG treatment for 72 h recovered L-EPCs’ proliferation and migration, as well as restored the phosphorylation level of Akt and eNOS blocked by high glucose condition.

**Conclusion:** EGCG accelerated reendothelialization in diabetic rabbits after carotid injury in part by reactivating the Akt/eNOS pathway, which might contribute to recovering proliferation and migration of L-EPCs impaired by high glucose.

## Introduction

Diabetes mellitus, characterized by high glucose condition, is one of the most common risks for cardiovascular disease. High glucose condition leads to endothelial dysfunction and reduces neovessel growth (Sheetz and King, 2002). Endothelial progenitor cells (EPCs), which were firstly found in human peripheral blood in the late 20th century, shows a potential to differentiate into mature endothelial cells and promotes reendothelization. Under many pathological conditions, EPCs participate in the process of neovascularization and endothelium self-repairing (Asahara et al., 1997, 1999). Recent studies reveal that late EPCs (L-EPCs) resemble endothelial cells, not only in morphology, growth characteristics, and cluster of differentiation molecules, but also in functional protein expressions, capability of incorporating into neovessels, and differentiating into endothelial cells (Iwakura et al., 2003; Hur et al., 2004). However, functions of L-EPCs are impaired in both diabetes and high glucose condition (Fadini et al., 2005).

Epigallocatechin gallate (EGCG) is the most abundant catechin in green tea and has proven benefits in maintaining vascular homeostasis in various diabetic models as a strong antioxidant. Polyphenols including EGCG could improve endothelial dysfunction in diabetes through up-regulation of endothelial nitric oxide synthase levels, increasing expression of vascular endothelial growth factor, inhibiting endoplasmic reticulum stress, inflammatory and oxidative stress (Suganya et al., 2016). Zhang et al show that EGCG promoted both proliferation and differentiation of mouse cochlear neural stem cells by activating the PI3K/Akt signaling pathway (Zhang et al., 2016). Our previous study demonstrates that activated phosphorylation of the PI3K/Akt/eNOS signal transduction pathway could improve the migration of L-EPCs (Zheng et al., 2007). Therefore, EGCG might also have positive effect on certain stem cells. We speculate that EGCG improving endothelial dysfunction in diabetes might be benefit from its effects on L-EPCs. High glucose condition inhibits L-EPCs’ proliferation, migration and promotes apoptosis through inactivating phosphorylation of the Akt/eNOS pathway, and consequently results in poor neovascularization. It is our hypothesis that EGCG could improve the repairing function of L-EPCs in high glucose condition. In this study, we evaluate the efficacy of EGCG pre-treated L-EPCs in improving reendothelialization in diabetic rabbits after carotid injury.

## Materials and Methods

### Isolation, Cultivation, Characterization of L-EPCs

Late endothelial progenitor cells were isolated, cultured and characterized according to the previously described methods (Hur et al., 2004). From each of male New Zealand white rabbits, 20 ml blood was obtained *via* ear vein. Peripheral blood mononuclear cells (PBMCs) were then isolated by density gradient centrifugation with Ficoll separating solution (Cedarlane Laboratories Ltd., ON, Canada). Cells were washed with M199 medium and re-suspended in 6 ml EGM-2MV (Lonza, Basel, Switzerland) containing 10% FBS, and then seeded in 3 wells of a fibronectin-coated six-well plate (2.5 μg/cm^2^). Non-adherent cells were removed after 2 days and the medium was replaced every 2 days thereafter. L-EPCs were passaged to a new 6-well plate at 80% confluence, typically after 2–3 weeks of the first passage. L-EPCs were then characterized as adherent cells double positive on Dil-acLDL-uptake and FITC-lectin binding by laser scanning confocal microscope, and were further documented by demonstrating the expression of CD34 (BD Bioscience, United States) and KDR (Miltenyi Biotec, Germany) by flow cytometry. For L-EPCs autotransfusion experiment, cells were labeled with CM-Dil (Invitrogen Technologies, United States) according to the previous study (Liu et al., 2011).

### Animals Experiments

#### Animals and Grouping

**FIGURE 1:**
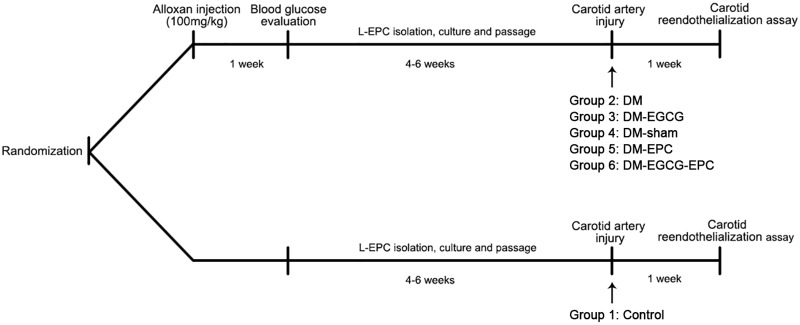
Timeline of *in vivo* experiments. Rabbits were randomized into six groups. Group 1 (Control): control rabbits given saline intragastrically after injured carotid artery operation. Group 2 (DM): diabetic rabbits given saline intragastrically after injured carotid artery operation. Group 3 (DM-EGCG): diabetic rabbits given EGCG 50 mg/kg/day intragastrically for 7 days after injured carotid artery operation. Group 4 (DM-sham): injured carotid artery of diabetic rabbits washed and then incubated 10 min by saline after injured operation. Group5 (DM-EPC): autologous L-EPCs transplanted into the area of injured carotid artery of diabetic rabbits after injured carotid artery operation. Group 6 (DM-EGCG-EPC): EGCG pre-treated autologous L-EPCs transplanted into the area of injured carotid artery of diabetic rabbits after injured carotid artery operation.

All animal experiments protocols were in accordance with the Declaration of Helsinki, and approved by the institutional Animal Care and Use Committee of Zhejiang University. Thirty-six male New Zealand white rabbits (2–3 kg) were provided by the Experimental Animal Center of Zhejiang University, China. All rabbits were maintained in individual cages (12 h light/12 h dark cycles) with normal diet. Rabbits were randomized into six groups (Figure 1). Each rabbit was performed carotid artery injured operation, and then L-EPCs were autologous transplantation pre-treated or not pre-treated with EGCG. Group 1 (Control) was control rabbits with saline intragastrically after operation. Group 2 (DM) and 3 (DM-EGCG) were diabetic rabbits with saline intragastrically after operation, or EGCG 50 mg/kg/day intragastrically for 7 days after operation. For Group 4 (DM-sham), injured carotid arteries of diabetic rabbits were washed and then incubated 10 min by saline after injured operation. For Group5 (DM-EPC) and Group 6 (DM-EGCG-EPC), autologous L-EPCs and EGCG pre-treated autologous L-EPCs were transplanted into the area of injured carotid artery of diabetic rabbits after procedure.

#### Diabetic Rabbit Model and Carotid Injury Model Establishing

Diabetic rabbit model was rendered by using a single dose of alloxan (100 mg/kg; Sigma-Aldrich, Canada) injection intraperitoneally. Animals were considered diabetic if the fasting blood glucose level was >15 mmol/L for 1 week after the injection. The diabetic rabbits were then given normal diet for 4–6 weeks with fasting blood glucose level checked once per week. Only rabbits with all-time fasting blood glucose level >15 mmol/L were used in the study (Pomaro et al., 2005). Diabetic rabbits were anesthetized by pentobarbital sodium (40 mg/kg) *via* intraperitoneal injection. Rabbit carotid injury was performed by using a 3.0 mm × 8 mm non-compliant balloon (Boston Scientific, United States) inflated and withdrawn three times in the carotid to denude the endothelium as described (Chen et al., 2010). Figure 1 shows the timeline of the *in vivo* experiment.

For the L-EPCs autologous transplantation experiment, cells of each rabbit were first cultured and pre-treated with or without EGCG (Purity ≥ 95%, Sigma, United States. 40 μM for 72 h) (Zhang et al., 2016). After that, a total of 1 × 10^6^ cells were resuspended in 200 μl of 4°C saline. Immediately after the balloon withdrawal, cells were injected into the carotid lumen, and were kept for 10 min before the incision was closed.

#### *In vivo* Reendothelialization Assay

Seven days after the carotid injury, areas of rabbit carotid reendothelialization assay were detected by injection with 2 ml of 5% Evans blue (Sigma-Aldrich, Canada) dissolved in saline via ear vein. One hour after the injection, animals were euthanized and carotid arteries were harvested, dissected out, opened longitudinally, and fixed with 4% paraformaldehyde (Sangon Biotech, Shanghai, China). The pictures were taken and the area of denudation was identified using the NIH Image J software by an investigator blinded to the group assignment. In CM-Dil labeled L-EPC auto-transfusion groups, the homing of transplanted EPCs to the site of injured vascular was analyzed using fluorescent microscopy (Olympus, Tokyo, Japan).

### Cell Experiments

Cell viability was assayed by a colorimetric procedure using the Cell Counting Kit-8 (Dojindo, Shanghai, China) according to the manufacturer’s protocol. Then, cells were randomized into four groups: control, mannitol, high glucose, and high glucose group with EGCG 40 μM treatment. For the high glucose group, cells were co-incubated with 30 mM glucose, whereas cells in the osmotic pressure control (mannitol group) were co-incubated with 30 mM mannitol.

#### *In vitro* L-EPCs Proliferation Assay

To detect the exact proliferation rates of L-EPCs an EDU (5-ethynyl-20-deoxyuridine) incorporation assay was executed with the Cell-Light^TM^ EdU *in vitro* Imaging Kit (Ribobio, Guangzhou, China) according to the manufacturer instructions. Briefly, cells at 70–80% confluence were treated with 50 μM EDU in EGM-2MV medium and then incubated for 2 h before fixation in 4% paraformaldehyde. After the EDU staining, cell nuclei were stained with Hoechst 33342 and observed with an inverted fluorescent microscope (Olympus, Tokyo, Japan). For each group, 6 random fields were photographed. The proliferation rate refers to the number of EDU stained cells divided by the number of Hoechst 33342 stained cells.

#### *In vitro* L-EPCs Migration Assay

Late endothelial progenitor cells migration assay was performed with a modified Boyden chamber assay as described previously (Qiu et al., 2009). Briefly, 5 × 10^4^ cells were loaded into the upper chamber of 8 μm pore size Millicell Culture Plate Insert (Millipore, Billerica, MA, United States) in 200 μl EGM2-MV supplemented with 1% FBS. Also 600 μl of 10% FBS EGM2-MV were loaded to the lower chamber. Cells were set to migrate for 3 h before fixation in 4% paraformaldehyde, and cells not migrated were removed from the upper chamber surface with cotton swabs. Migrated cells on the lower surface of the filter were stained with 4′6-diamidino-2-phenylindole (DAPI) (Beyotime, Shanghai, China). Cells were examined under a fluorescent microscope (Olympus, Tokyo, Japan) at a 400 × total magnification and 6 random fields were photographed for each sample. The average number of migrated cells in the 6 random fields was computed and used as the migration number for the group.

### Quantitative RT-PCR

Total RNA of the cultured L-EPCs was extracted with standard TRIZOL (Invitrogen, Carlsbad, CA, United States) method. cDNA synthesis was performed with 1 μg of total RNA using the PrimeScript^TM^ RT-PCR Kit (Takara, Shiga, Japan) following the manufacturer’s instructions. Real-time PCR was performed on the ABI 7500 cycler (Applied Biosystems, CA, United States), using the Soso fast Eva Green Supermix (Bio-Rad, Hercules, CA, United States) in compliance with the manufacturer’s protocol. β-actin was used as endogenous controls for mRNAs expression. Primers of Akt, eNOS, and β-actin were synthetized by Invitrogen (Carlsbad, CA, United States).

### Western Blots and Antibodies

Cells in a 6-well plate were scraped in a RIPA lysis buffer (Beyotime, Shanghai, China) supplemented with 1 mM PMSF. Proteins (20 μg) were separated on 10% SDS-polyacrylamide gels and were electro-transferred to polyvinylidene difluoride (PVDF) membranes (Bio-Rad, Hercules, CA, United States). After a blocking incubation with 5% milk-TBST, the membranes were incubated overnight in primary antibodies at appropriate dilutions, followed by 1 h incubation in a secondary antibody conjugated to horseradish peroxidase (1:10000 dilution). After incubations in an enhanced chemiluminescence reagent (Amersham, Haemek, Israel), images were captured and analyzed on the LAS-4000 image reader system (Fujifilm, Tokyo, Japan).

Anti-Akt/p-Akt, anti-eNOS/p-eNOS, and anti-β-actin antibodies for western blotting were provided by Cell Signaling Technology (Beverly, MA, United States) and Santa Cruz Biotechnology (Dallas, TX, United States), respectively.

### Statistical Analysis

Data were presented as means ± SEM. Analyses were conducted with SPSS 18.0 software using unpaired Student’s *t*-test for comparisons between two groups and one-way ANOVA for multi-group comparisons. *P* < 0.05 was considered as statistically significant.

## Results

### L-EPC Characterization

**FIGURE 2:**
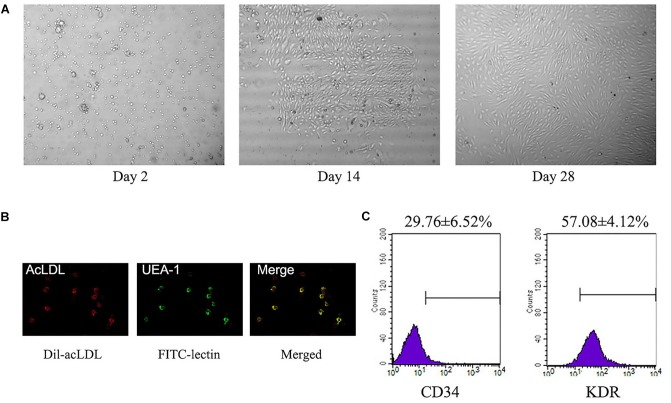
Identification of L-EPCs. **(A)** The panel shows the sequential changes of L-EPCs. All the pictures were taken under ×100 magnification. **(B)** The L-EPCs shows both Dil-uptaken (red) and lectin binding (green) ability (× 400 magnification). **(C)** Flow-cytometry reveals the L-EPCs expressed abundant CD34 and KDR molecule. Numbers are presented as Mean ± SEM. Percentage of positive cells for all experiment is determined by comparison with corresponding negative control labeling.

A total of about 3 × 10^6^–1 × 10^7^ of PBMCs was isolated from each 20 ml blood sample. These PBMCs were cultured for 14∼28 days and displayed abundance of attached cobblestone morphology cells (Figure 2A). Most of the cells were double-positive for Dil-acLDL uptake and FITC–lectin binding (Figure 2B). There were 29.8 ± 6.5% CD34^+^ positive cells, and 57.1 ± 4.1% KDR positive cells (Figure 2C).

### EGCG Intragastric Administration Improving Reendothelialization of Diabetic Rabbit Carotid

**FIGURE 3:**
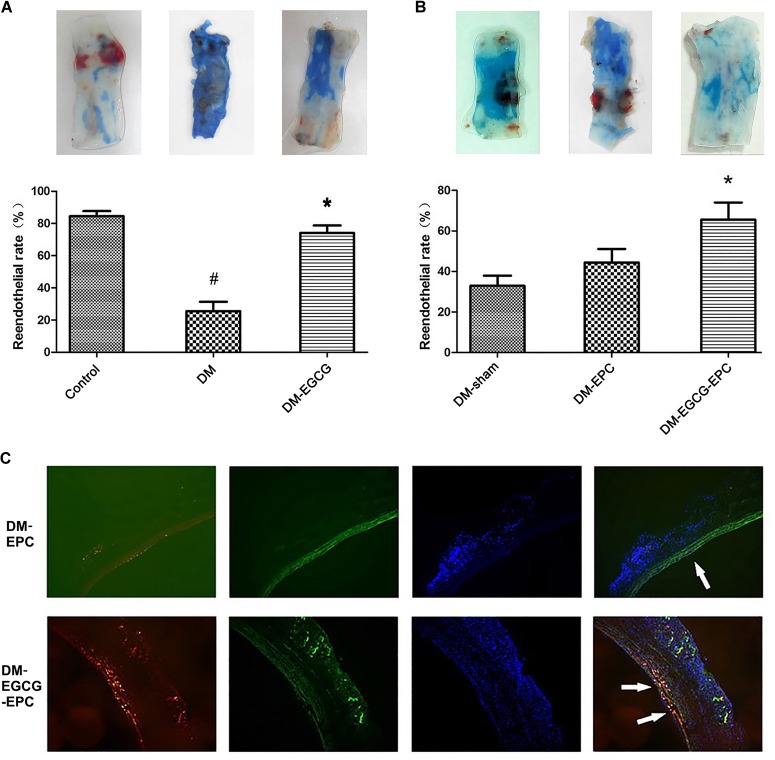
Reendothelialization of injured carotid arteries is promoted by EGCG. **(A)**Reendothelialization of injured carotid arteries is promoted by intragastrically-given of EGCG. The blue area, which was rendered by Evans Blue, represent the area of carotid artery where lack of endothelium. The control group shows the best reendothelialization, whereas diabetes rabbits show very poor reendothelialization. After intragastrically given EGCG for 7 days, the reendothelialization rates risen. The number of rabbits in each group was 6. DM: diabetic rabbits were given with saline intragastrically after injured carotid artery operation. DM-EGCG: diabetic rabbits were given with EGCG 50 mg/kg/day intragastrically for 7 days after injured carotid artery operation. ^#^*P* < 0.05 and ^∗^*P* < 0.05 compared to the control group or the diabetes group, respectively. **(B)** EGCG pre-treated autologous L-EPCs transfusion improved diabetic rabbits carotid reendothelialization. The panel shows the representative reendothelialization photographs of the 3 groups (*n* = 6 for each group). After autologous L-EPCs transplantation, diabetic rabbits’ carotid reendothelialization was improved but no statistical difference; however, by transfusion autologous EGCG pre-treated L-EPCs, the reendothelialization was significantly improved. DM-sham: injured carotid artery of diabetic rabbits were washed and then incubated 10 min by saline after injured operation. DM-EPC: autologous L-EPCs were transplanted into the area of injured carotid artery of diabetic rabbits after injured carotid artery operation. DM-EGCG-EPC: EGCG pre-treated autologous L-EPCs were transplanted into the area of injured carotid artery of diabetic rabbits after injured carotid artery operation. ^∗^*P* < 0.05 compared to the diabetic sham group. **(C)** Representative figure of autologous L-EPC tracking *in vivo* under fluorescent microscope. Picture shows the extent to which L-EPCs are involved in reendothelialization 7 days after carotid artery injury. CM-DiI-labeled L-EPCs (red) are attached to endothelium (FITC-lectin-stained, green). Nuclei of L-EPC were stained with DAPI (blue).

There was no statistical difference on rabbit weight and age among all the three groups (data not shown). Diabetic model was rendered successfully by using an alloxan method. The average fasting blood glucose level was 5.6 ± 0.5 mmol/L and 25.6 ± 1.2 mmol/L before and 1 week later after alloxan injection, respectively. Compared with the control group, diabetic rabbits showed poor reendothelialization ratios after carotid injury (25.6 ± 5.9% vs. 84.6 ± 3.1%, *P* < 0.001), as shown in the Evans blue dyeing assay (Figure 3A). Diabetic rabbits with intragastrically given EGCG (50 mg/kg/d) for 7 days showed similar reendothelialization rates of injured carotid comparing with the control group without diabetes (74.2 ± 4.6% vs. 84.6 ± 3.1%, *P* = 0.284; Figure 3A).

### EGCG Pre-treated L-EPCs Autologous Transfusion Accelerating Diabetic Rabbit Carotid Reendothelialization

To identify whether EGCG benefits carotid reendothelialization through improving the functions of L-EPCs, we transfused autologous L-EPCs to the carotid injured rabbits. Compared with diabetic sham-operated group, diabetic autologous L-EPCs transplantation locally to the injured carotid artery showed an accelerated trend of the reendothelialization process, but statistical difference was not found (44.4 ± 6.8% vs. 32.9 ± 5.0%, *P* = 0.485). After EGCG pre-treatment for 72 h, autologous L-EPCs transfusion strongly improved the reendothelialization of diabetic rabbits (65.6 ± 8.5% vs. 32.9 ± 5.0%, *P* = 0.011; Figure 3B). Moreover, autologous L-EPCs transfusion group (EGCG pre-treated) displayed clearly more CM-Dil labeled L-EPCs attached to the endothelium, compared with the non-pre-treated ECGG group (Figure 3C).

### Improved L-EPCs’ Proliferation by EGCG in High-Glucose Environment

**FIGURE 4:**
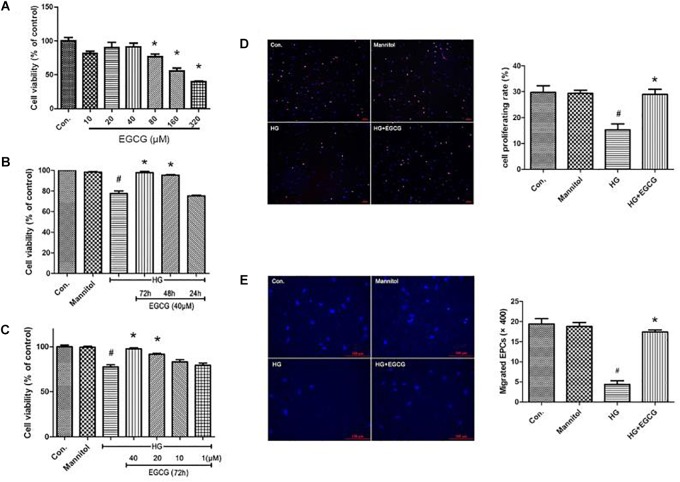
EGCG improves L-EPCs proliferation and migration under high glucose condition. **(A)** CCK-8 test reveals that up to 40 μM EGCG shows no poisonousness to L-EPCs, the typical IC50 of the chemical is 320 μM. ^∗^*P* < 0.05 compared to control group. **(B,C)** L-EPCs incubated in high glucose environment (30 mM) were co-incubated under different concentrations of EGCG (1–40 μM) for different time (24–72 h). Histogram reveals that high glucose treatment for 72 h directly impairs the proliferation of L-EPCs, this impairment could be block by EGCG in a time and dose dependent manner. ^#^*P* < 0.05 compared to the control group, ^∗^*P* < 0.05 compared to the high glucose (HG) group. **(D)** EDU incorporation assay (× 100 magnification). In the image overlay, the purple nuclei stained with EDU, indicates proliferating cells, while the blue nuclei are Hoechst 33342 stained, ^#^*P* < 0.05 compared to the control group, ^∗^*P* < 0.05 compared to the HG group. **(E)** Boyden chamber assay, pictures reveals 40 μM EGCG incubated for 72 h could reversed the down-regulation of L-EPCs’ migration induced by high glucose. The magnification is × 400. ^#^*P* < 0.05 compared to the control group, ^∗^*P* < 0.05 compared to the HG group.

Late endothelial progenitor cells viability was determined by a CCK-8 test. We first examined the toxicity of EGCG to L-EPCs. Total confluent L-EPCs cultured in 96-well plate were added different concentrations of EGCG and their absorbance at 460 nm was examined. As shown in Figure 4A, up to 40 μM concentration of EGCG showed no poisonousness to L-EPCs. The typical IC50 of EGCG is 320 μM. High glucose treatment for 72 h directly impaired the proliferation of L-EPCs, while this impairment was greatly blocked in a time-and-dose dependent manner by EGCG. EGCG treatment of 40 μM for 72 h could most effectively recover the proliferation of L-EPCs impaired by high glucose (Figures 4B,C). EDU incorporation assay also demonstrated the benefit of EGCG in improving L-EPCs’ proliferation (Figure 4D).

### EGCG Abrogates High Glucose Mediated Inhibition on L-EPCs’ Migration *in vitro*

The mobility of L-EPCs was examined by a Boyden chamber assay. High glucose directly inhibited L-EPCs’ migration to one quarter of control group, but this inhibition could be reversed mostly by an EGCG contained culturing environment (40 μM for 72 h; Figure 4E).

### EGCG Restores the Level of p-Akt and p-eNOS Blocked by High Glucose

**FIGURE 5:**
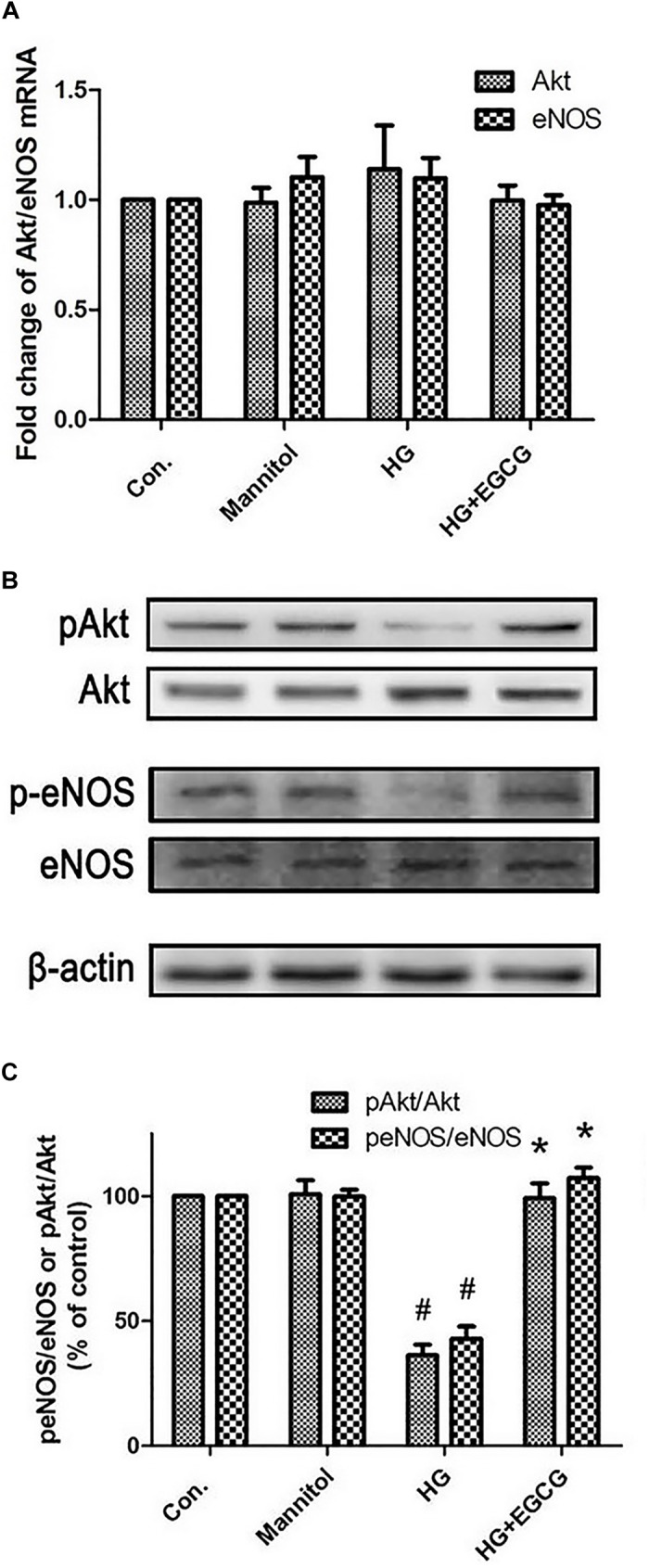
EGCG re-activates Akt-eNOS pathway blocked by high glucose treatment. **(A)** RT-PCR shows the relative expression of Akt and eNOS mRNA, which were not changed significantly between the different groups. **(B,C)** Western blot reveals the phosphorylation of Akt and eNOS were blocked by HG treating, and recovered by 40 μM EGCG co-incubation for 72 h. ^#^*P* < 0.05 compared to the control group, ^∗^*P* < 0.05 compared to the HG group.

The mRNA level of both Akt and eNOS remained unmodified by 72 h high glucose treatment. Also the EGCG treatment did not affect the Akt and eNOS mRNA level (Figure 5A). Compared with the control group, high glucose blocked the phosphorylation of Akt and eNOS in diabetic rabbits by half as shown in the western blot test, and the EGCG treatment restored the level of p-Akt and p-eNOS completely (Figures 5B,C).

## Discussion

The primary cause of vascular complications in diabetes mellitus is hyperglycaemia, associated with endothelial dysfunction and impaired neovascularisation. Clinical studies have revealed that diabetic patients usually have both low numbers and low activities of EPCs (Fadini et al., 2007; Makino et al., 2008). In diabetic patients, L-EPCs, which have more important effect than early-EPCs during repair of artery injury, exhibit impaired abilities of differentiation, proliferation, adhesion and migration, tubulisation, secretion, mobilization, and homing (Chen et al., 2007). Other studies have reported that high glucose condition, similar to diabetes mellitus, could directly suppress the proliferation, migration and Akt activity of L-EPCs (Nakamura et al., 2011).

Epigallocatechin gallate is known as an antioxidant, but recent investigations have revealed other direct actions independent from it, such as anti-hypertrophy, anti-inflammatory, anti-myocardial infarction and anti-atherosclerosis (Kim et al., 2014; Rashidi et al., 2017; Eng et al., 2018).

In our study, 50 mg/kg treatment is selected as therapeutic dose according to study of Hong’s team (Hong et al., 2002). Other studies show that 25 mg/kg, 50 mg/kg, and 100 mg/kg EGCG treatments are also therapeutically effective in rabbit (Li et al., 2009). In one study (Wu et al., 2017), intragastrical EGCG (100 mg/kg/day) in diabetic rats is effective to myocardial ischemia/ reperfusion injury through ameliorating post-ischemic cardiac dysfunction, decreasing the myocardial infarct size, apoptosis, and cardiac fibrosis.

Our previous studies show, neonatal rat cardiomyocytes and human endothelial cells are cultured with 40 μM EGCG treatment for 24 h (Yu et al., 2010; Zhao et al., 2011). In this study, six different EGCG concentrations from 10 to 320 μM and three different durations (24, 48, and 72 h) are tested. Among those, 40 μM EGCG treatment for 72 h displays the optimal improvement on the proliferation and migration of L-EPCs.

Here, we first observed that EGCG could improve reendothelialization of injured carotid artery of diabetic rabbits. To find out whether L-EPCs is involved, subsequent EGCG pre-treated L-EPCs autotransfusion experiments were performed. We find that the EGCG (40 μM for 72 h) pre-treated L-EPCs show significantly improved maturation of endothelial cells than L-EPCs only, consequently ameliorate reendothelialization of the injured artery in diabetic rabbits. Therefore, EGCG might facilitate L-EPCs differentiate to endothelial cells and improve the reendothelialization of diabetic rabbits’ injured carotid artery. To find out the underlined mechanism, the mobility and proliferation of L-EPCs are examined. High glucose directly inhibits L-EPCs’ proliferation and migration, but this inhibition could be significantly reversed by an EGCG-contained culturing environment. Finally, our study shows that EGCG improves the reendothelialization of diabetic rabbits, potentially by promoting the proliferation and migration of L-EPCs under high glucose condition.

Several signal pathways have been reported to play important roles under high glucose condition induced suppression of EPCs (Dimmeler et al., 2000; Kuki et al., 2006). Among them, the Akt/eNOS pathway is generally considered to be the most important one. Expression and phosphorylation of Akt and endothelial nitric oxide synthase (eNOS) are known to be essential for EPCs and endothelial cells’ migration and angiogenesis (Dimmeler et al., 2000; Aicher et al., 2003). However, this pathway is suppressed under high glucose condition (Chen et al., 2007; Fadini et al., 2007).

In endothelial cell lines and myocardiocytes, EGCG also influences the phosphorylation of the Akt/eNOS pathway (Kurita et al., 2013). Liu’s study shows that EGCG protects human umbilical vein endothelial cells against apoptosis by modulating mitochondrial-dependent apoptotic signaling and the PI3K/Akt/eNOS signaling pathway. EGCG enhances the protein ratio of p-Akt/Akt, eNOS activation, and nitric oxide formation in Hcy-induced injured cell (Liu et al., 2017). Xuan and colleagues report EGCG protection of rat’s heart against ischemia/reperfusion injury through the PI3K/Akt pathway-mediated inhibition of apoptosis and restoration of the autophagic flux in cardiomyocytes (Xuan and Jian, 2016). In Zeng’s study, 10 μM EGCG also protects H9c2 cells through activating the PI3K/Akt pathway and downregulating TNF-α, IL-6, and IL-8 levels (Zeng and Tan, 2015). Therefore, we focus on whether EGCG benefits L-EPCs through the Akt/eNOS signal pathway.

In present study, high glucose condition (30 mmol/l) impairs the phosphorylation of Akt/eNOS protein even if the mRNA levels of Akt and eNOS remain unchanged, whereas EGCG treatment restores the level of p-Akt and p-eNOS close to the control group in our experimental condition. EGCG, at least partially, blocks the impairment of high glucose to L-EPCs through reactivating the Akt/eNOS pathway. However, the exact mechanism of how EGCG promotes the phosphorylation of Akt and eNOS need to be investigated in future studies.

Corbi and his team display that rabbits taking phenolic plant extracts orally induced Sirt1 activity and increased antioxidant levels in the rabbit’s heart and liver (Corbi et al., 2018). EGCG (50 mg/kg) can alleviate liver injury and oxidative stress in hyperlipidaemic rats through activating the SIRT1/FOXO1 signaling pathway, regulating the SREBP-2 protein, and inhibiting hepatic cholesterol synthesis (Li and Wu, 2018). Study from Conti and the group about human endothelial cells also confirms that the pathway of Sirt1 is a critical regulator of oxidative stress response and cellular lifespan (Conti et al., 2015). And other investigation proves phytochemicals may act as positive modulators of neuroinflammatory events (Corbi et al., 2016). Therefore, EGCG might have acted on multiple pathways to improve endothelial function and the whole anti-oxidative environment, which contribute to increased reendothelialization in diabetic rabbits. Whereas, this study is only designed to investigate the Akt/eNOS pathway and examines on other pathways and antioxidant levels of important organs in *ex vivo* are warranted.

There are several deficiencies in our study need to be improved in the future study, such as relatively small sample size, no specific inhibitor against Akt/eNOS signal pathway, and lack of whole anti-oxidative ability evaluation. But, to our knowledge, present study is the first one showing that EGCG improves the reendothelialization of carotid artery in diabetic rabbits, potentially by promoting the proliferation and migration of L-EPCs under the high glucose condition. The activations of the phosphorylation of Akt and eNOS play an important role during this process. What is noteworthy is that concentrations used in our *in vivo* study are supramaximal to average daily tea consumption. Data displayed that when human average daily tea consumption almost contains 1600 mg EGCG, the maximum blood concentration is 7.4 μM according to bioavailability. The maximum blood concentration is 17.5 μM after 50 mg/kg EGCG oral administration according to other rabbits’ bioavailability experiments. Therefore, our experimental results should not be made an analogy to the real world simply. In conclusion, our study demonstrates the efficacy of EGCG on endothelial dysfunction in carotid artery caused by diabetics, and the potential of EGCG in treating diabetic peripheral atherosclerotic disease in clinical application.

## Author Contributions

HH and DH designed and performed the study. HH, C-yJ, and DH wrote the manuscript with input from all authors. C-yJ, X-kB, and LY designed and performed the animal experiments. S-jX, Y-bZ, and M-hW performed the cell growth and *in vivo* studies. C-yJ and Y-xS performed the analytic calculations and statistical analysis. All authors provided critical feedback and helped to shape the research, analysis, and manuscript.

## Conflict of Interest Statement

The authors declare that the research was conducted in the absence of any commercial or financial relationships that could be construed as a potential conflict of interest.
